# Fumonisin B1 Exerts Immunosuppressive Effects Through Cytoskeleton Remodeling and Function Attenuation of Mature Dendritic Cells

**DOI:** 10.3390/ijms26072876

**Published:** 2025-03-21

**Authors:** Yanqin Yu, Xue Zhao, Yao Cheng, Guofu Shang, Kaiyi Tang, Yun Wang, Xiaoyan Peng, Sha Ou, Zuquan Hu

**Affiliations:** 1 Immune Cells and Antibody Engineering Research Center in University of Guizhou Province, School of Basic Medical Sciences/School of Biology and Engineering (School of Modern Industry for Health and Medicine), Guizhou Medical University, Guiyang 550025, China; yuyanqin@stu.gmc.edu.cn (Y.Y.); 2022110111159@stu.gmc.edu.cn (Y.C.); shangguofu@stu.gmc.edu.cn (G.S.); tangkaiyi@gmc.edu.cn (K.T.); wangyun@gmc.edu.cn (Y.W.); 2 Guizhou Provincial Engineering Research Center for Smart Biomaterials, Key Laboratory of Biology and Medical Engineering, Guizhou Medical University, Guiyang 550025, China; 15085981741@163.com (X.Z.); pengxiaoyan@gmc.edu.cn (X.P.); 3 Guizhou Key Laboratory of Microbio and Infectious Disease Prevention & Control, Guizhou Medical University, Guiyang 550025, China

**Keywords:** fumonisin, dendritic cells, mechanical properties, cytoskeleton, immunosuppression

## Abstract

Fumonisin B1 (FB1) is one of the most toxic mycotoxins and is harmful to humans and animals due to its hepatotoxicity, immunotoxicity and carcinogenicity. However, the mechanism of its immunosuppressive effect is still under investigation. Dendritic cells (DCs) are the most potent professional antigen-presenting cells, and their differentiation, maturation and immunomodulatory functions are closely related to the immunotoxicity of certain mycotoxins. Migratory capacity is a prerequisite for mature DCs (mDCs) to move and present antigens in secondary lymphoid tissue, whereas the mechanical properties and cytoskeletal structure are critical for their migration and immune functions. Therefore, the effects of FB1 on the cell viability, mechanical characteristics, cytoskeletal structure and its binding proteins, migration, co-stimulatory molecules and the immune functions of mDCs were investigated to explore the potential mechanisms of immunotoxicity. The results showed that FB1 could impair the chemotactic migratory capability, the expression of co-stimulatory molecules and the ability of DCs to stimulate T cell proliferation. Further analyses elucidated that the mechanical properties of mDCs were changed, the cytoskeletal structures were reorganized and the expressions of cytoskeleton-binding proteins were regulated. In conclusion, the attenuated migration and immune functions of mDCs caused by FB1 may be related to their altered mechanical properties and cytoskeleton remodeling, which may be one of the action modes for FB1 to exert its immunosuppressive effect.

## 1. Introduction

Fumonisin B1 (FB1) is a toxic metabolite mainly produced by *Fusarium verticillioides* and poses a health risk to animals and humans [1]. FB1 is classified as a Group 2B carcinogen by the International Agency for Research on Cancer (IARC), indicating its potential carcinogenicity to humans [2]. Chronic exposure to FB1-contaminated food is associated with an increased risk of kidney disease, liver cancer and esophageal cancer, which may be linked to its immunotoxic effects [2,3]. Therefore, elucidating the immunotoxic mechanisms of FB1 is crucial for the development of strategies to treat and prevent FB1-induced mycotoxicosis. Current studies indicate that the toxicological effects of FB1 are primarily due to its competitive inhibition of ceramide synthetase and the disruption of normal sphingolipid metabolism, leading to oxidative stress and endoplasmic reticulum stress [1,2]. Additionally, FB1 is shown to inhibit the zymosan-induced formation of neutrophil extracellular traps (NETs) in chicken neutrophils by blocking the burst of reactive oxygen species (ROS) and the release of histone H3 and neutrophil elastase, resulting in immunosuppression [4]. However, the precise mechanisms underlying FB1’s immunotoxicity are still under investigation from multiple perspectives.

Dendritic cells (DCs) are the most powerful professional antigen-presenting cells bridging innate and adaptive immunity. Upon stimulation by foreign antigens or inflammatory cytokines, immature DCs (imDCs) migrate from peripheral tissues to secondary lymphoid organs, where they ultimately differentiate into mature DCs (mDCs) [5]. During this maturation, the upregulation of major histocompatibility complex (MHC) molecules and cell surface co-stimulatory molecules provides the primary and secondary signals required for the activation of naive T cells. These signals determine antigen specificity and the intensity of T cell activation, respectively [6]. Given their critical roles, DCs can serve as a cell model to compensate for the insufficiency of existing immunotoxicity evaluation. Previous studies demonstrated that FB1 can not only reverse morphological changes but also suppress the lipopolysaccharide (LPS)-induced expressions of some surface molecules, cytokines and chemokines in human DCs and/or murine bone marrow-derived dendritic cells (BMDCs) [7,8]. These findings suggest that the immunosuppressive effects of FB1 are closely associated with the interfered immune functions of DCs, such as antigen uptake, processing and presentation. Certainly, the precise mechanisms underlying the effects of FB1 on DCs require further elucidation.

The mechanical properties and cytoskeleton structure of cells are integral to elucidating the relationship between cell structure and function [9,10,11,12,13]. Specifically, the cytoskeleton is a key determinant of cellular mechanical properties [14]. Alterations in these properties and the cytoskeleton can directly influence cell deformability, adhesion, migration and interaction with the extracellular matrix [15,16,17,18,19]. For instance, recent findings have indicated that the maturation of BMDCs leads to increased cell stiffening and reduced nucleus mobility [20]. Additionally, our prior research showed that vascular endothelial growth factor (VEGF) can impair the motility and immune functions of mDCs by modulating their biophysical properties and cytoskeleton structure via the VEGFR2-RhoA-cofilin1 pathway [21,22]. However, it remains unclear whether the dysfunction of mDCs induced by FB1 is associated with the changes in mechanical properties, cytoskeleton remodeling and migration ability. Therefore, analyzing the mechanical characteristics, migration capacity and antigen presentation function of mDCs, as well as elucidating the action model of FB1, is of significant importance for a comprehensive understanding of the immunotoxicity mechanism of fumonisins from an interdisciplinary perspective.

## 2. Results

### 2.1. FB1 Reduces Viability but Does Not Induce Apoptosis of mDCs

**Figure 1:**
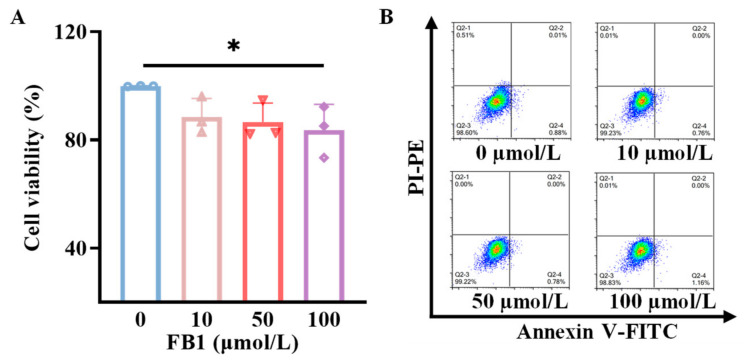
The toxicity of FB1 on mDCs. (**A**) CCK8 detection of the effect of FB1 on the cell viability of mDCs. (**B**) Flow cytometry detection of the effect of FB1 on the apoptosis of mDCs. Compared with the untreated FB1 group, * *p* < 0.05.

To elucidate whether the effects of FB1 on mDCs derived from murine bone marrow are related to the alterations in cell viability, the viabilities of mDCs were assessed using a CCK-8 detection kit following treatment with 10, 50 and 100 μmol/L FB1. As illustrated in Figure 1A, treatment with FB1 led to a dose-dependent decrease in cell viability, with a significant reduction observed only at the highest concentration of 100 μmol/L (*p* < 0.05). Additionally, apoptosis was evaluated using FITC-Annexin V and propidium iodide (PI) staining, followed by flow cytometry analysis. The results indicated that the apoptosis rates of mDCs remained largely unchanged following 48 h treatment with different concentrations of FB1 (Figure 1B).

### 2.2. FB1 Inhibits the Maturation and Immune Function of mDCs

**Figure 2:**
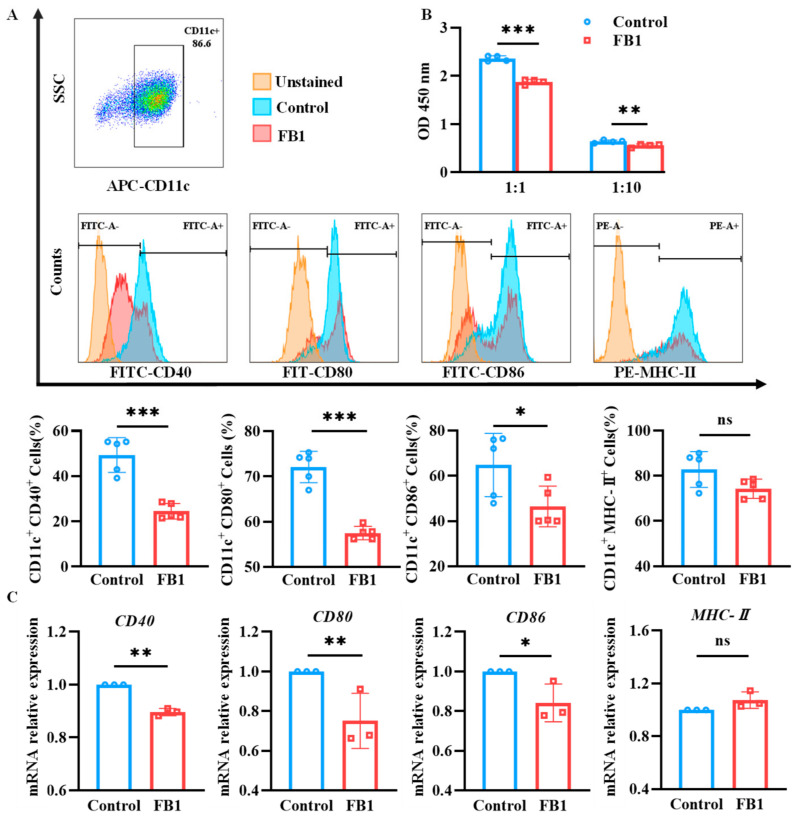
The expressions of key surface molecules on mDCs and their antigen presentation ability after FB1 exposure. (**A**) Flow cytometry detection of the CD40, CD80, CD86 and MHC-II expression on mDCs. (**B**) CCK8 detection of the antigen presentation ability of mDCs. (**C**) RT-qPCR detection of the *CD40*, *CD80*, *CD86* and *MHC-II* expression on mDCs. The *y*-axis represents the mRNA expression levels relative to the reference gene *GAPDH*. Compared with the control group, *** *p* < 0.001, ** *p* < 0.01 and * *p* < 0.05, and ns represents no significant difference.

After exposure to 50 μmol/L FB1 for 48 h, the cells were subjected to a labeling procedure using APC-conjugated anti-mouse CD11c antibody in combination with FITC-conjugated anti-mouse CD40, CD80 and CD86 antibodies, or PE-conjugated anti-mouse MHC-II antibody. The resulting fluorescent signals were detected by flow cytometry. Additionally, total RNA was extracted from the cells using the Trizol method, and reverse transcription was performed to generate cDNA. Subsequently, real-time quantitative PCR (RT-qPCR) was used for analyzing the mRNA relative expression levels of *CD40*, *CD80*, *CD86* and *MHC-II* molecules. The data revealed that the expression levels of CD40, CD80 and CD86 were significantly downregulated compared to the control group (*p* < 0.05) (Figure 2A,C). Concurrently, the mixed lymphocyte reaction assay showed that the ability of mDCs to activate T lymphocyte proliferation was diminished after treatment with 50 μmol/L of FB1 (*p* < 0.05) (Figure 2B). In contrast, there was no significant difference in MHC-II molecule expression (*p* > 0.05).

### 2.3. FB1 Diminishes the Chemotactic Migration Ability of mDCs

**Figure 3:**
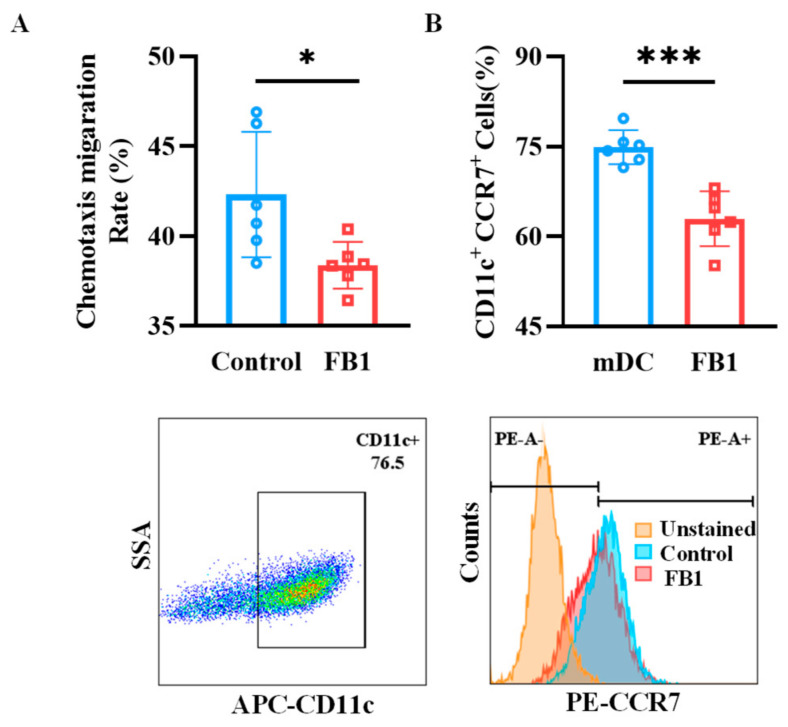
The chemotactic migration ability of mDCs after exposure to FB1. (**A**) The Transwell system investigated the CCL19 chemotactic migration ability of the mDCs. (**B**) Flow cytometry detection of the mDCs’ protein expression of CCR7. Compared with the control group, *** *p* < 0.001 and * *p* < 0.05.

Chemotactic migration is essential for mDCs to migrate and fulfill their immune functions. In this study, the Transwell system was employed to evaluate the migratory capacity of mDCs. The results indicated that the CCL19-dependent chemotactic migration of mDCs treated with FB1 was significantly reduced compared to the control group (*p* < 0.05) (Figure 3A). Consistent with this finding, the protein expression of the CCR7 receptor was downregulated in the FB1-treated group (Figure 3B).

### 2.4. FB1 Changes the Biophysical Characteristics and Morphology of mDCs

**Figure 4:**
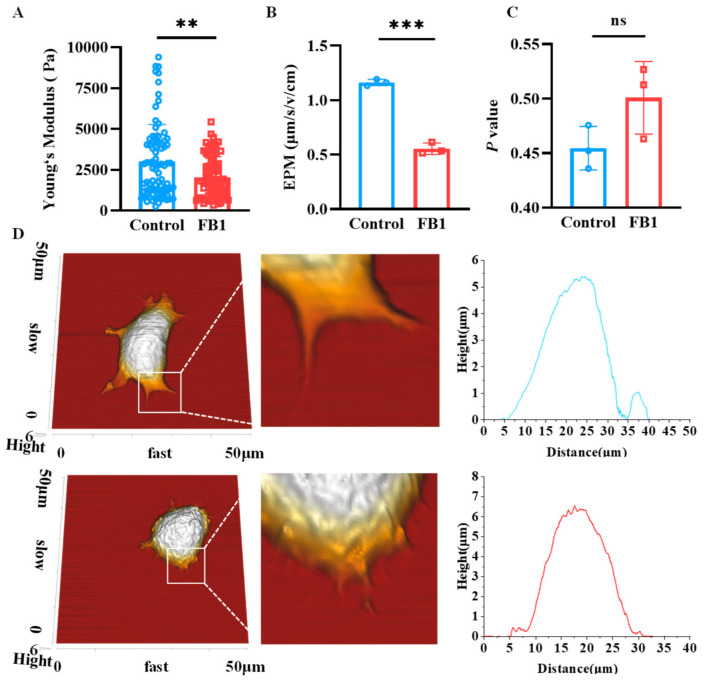
The mechanical characteristics of mDCs after FB1 exposure. (**A**) Young‘s modulus of the mDCs investigated by AFM; (**B**) electrophoretic mobility; (**C**) membrane fluidity and (**D**) AFM scanning of cell morphology. Compared with the control group, *** *p* < 0.001 and ** *p* < 0.01, and ns represents no significant difference.

The mechanical properties of cells could reflect the relationship between their structure and functions, and thus, the effects of FB1 on Young’s modulus, electrophoretic mobility (EPM) and membrane fluidity of mDCs were analyzed. The results revealed that both Young’s modulus and EMP significantly reduced compared to the control group (*p* < 0.01) (Figure 4A,B). However, there was no significant difference in the fluidity of the cell membrane lipid bilayer molecules (Figure 4C). In addition, the cell morphology was scanned by Atomic Force Microscopy (AFM) and the results showed that the morphology of the FB1-treated mDCs changed obviously, especially the filopodia, which was shorter than in the control group (Figure 4D).

### 2.5. FB1 Disrupts the F-Actin Structure of mDCs

**Figure 5:**
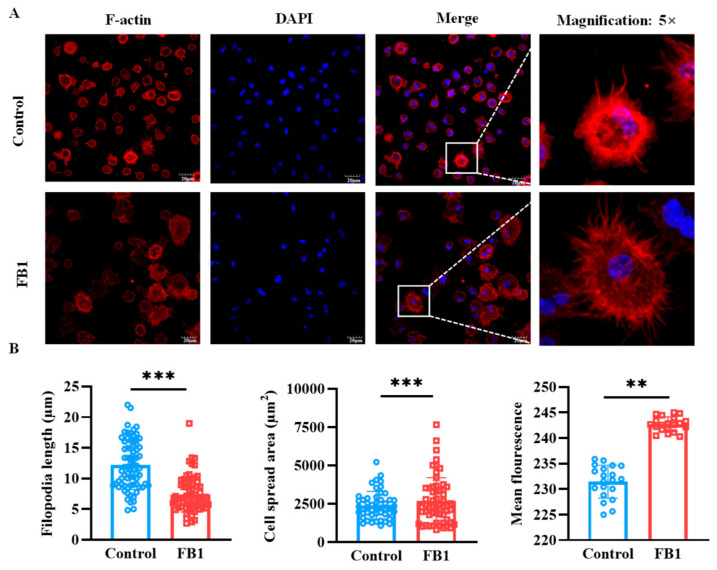
The cytoskeleton structural changes in mDCs induced by FB1 under laser confocal microscopy tomography. (**A**) Confocal microscopic analysis of F-actin organization of mDCs after treatment with FB1. (**B**) The change in filopodia length, cell spread area and F-actin expression of mDCs. Compared with the control groups, ** *p* < 0.01 and *** *p* < 0.001.

The cytoskeleton, a dynamic network of protein filaments, is pivotal in orchestrating cytoskeletal remodeling and shaping cellular morphology. To investigate the effects of FB1 on the morphology of mDCs, laser confocal microscopy tomography was utilized to provide a detailed visualization of the cytoskeletal architecture within mDCs following FB1 treatment. The results demonstrated that the F-actin structure within mDCs exposed to FB1 underwent significant reorganization, as evidenced by distinct alterations in filament length, cell spread area and the mean fluorescence of F-actin (Figure 5A and Figure S1). This observation was corroborated by AFM scanning, which provided complementary insights into the nanoscale topography of the cell surface. Specifically, the surface area and mean fluorescence of FB1-treated mDCs markedly increased compared to the controls (*p* < 0.01), suggesting substantial morphological changes at the cellular periphery (Figure 5B). Moreover, the length of the filopodia in FB1-treated cells significantly decreased (*p* < 0.01), indicating reduced cytoskeletal dynamics and potential alterations in cell motility. Collectively, these results highlight the profound impact of FB1 on the cytoskeletal integrity and morphology of mDCs, which may underlie the compromised migratory capacity and immune function of these cells.

### 2.6. FB1 Regulates the Cytoskeleton-Binding Protein Expression of mDCs

**Figure 6:**
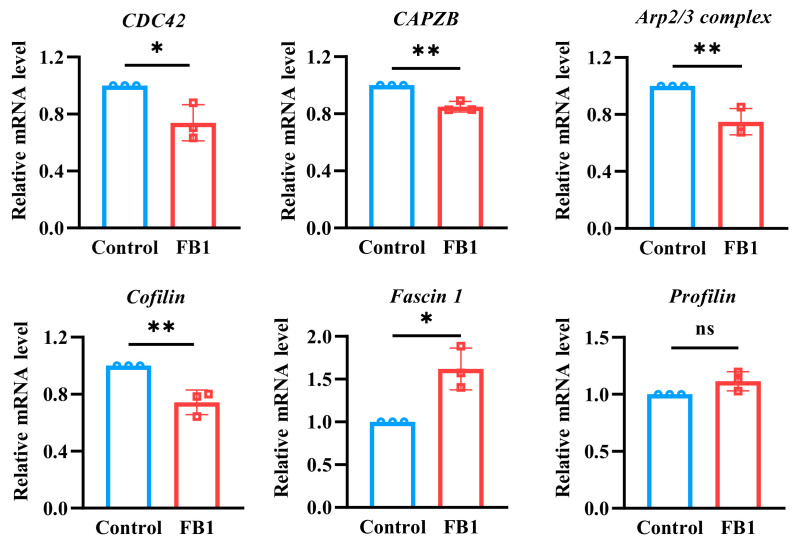
The mRNA expression levels of cytoskeleton-binding proteins were detected by RT-qPCR detection. The *y*-axis represents the mRNA expression levels relative to the reference gene *GAPDH*. Compared with the control groups, * *p* < 0.05 and ** *p* < 0.01, and ns represents no significant difference.

Due to the fact that cytoskeleton-binding proteins are closely related to cell morphology and cell motility, the mRNA expression levels of some main cytoskeleton-binding proteins were detected by real-time quantitative PCR (RT-qPCR) (Figure 6). The results showed that the mRNA expression levels of cell division cycle 42 (*CDC42*), capping actin protein of muscle Z-line subunit beta (*CAPZB*), *Arp2/3 complex* and *Cofilin* cytoskeleton-binding proteins were downregulated, while the mRNA expression levels of *Fascin1* were upregulated after FB1 exposure (*p* < 0.05). However, there was no significant difference in the mRNA expression of *Profilin*. Moreover, Cofilin dynamically regulated the cytoskeleton by cutting actin filaments and promoting actin depolymerization and repolymerization. This process is critical for cell morphology, polarity, migration and motility. Therefore, to further elucidate the effects of FB1 on cytoskeleton regulation, changes in Cofilin1 protein expression were examined by Western blot analysis. The results demonstrated that FB1 exposure significantly inhibited Cofilin1 protein expression (Figure S2).

## 3. Discussion

FB1 is a mycotoxin to induce immunotoxicity and carcinogenicity in various species, including poultry, swine, mice and bovine. Despite extensive research, the precise mechanisms underlying FB1-induced immunosuppression remain unclear. In this study, we explored the effects of FB1 on the mechanical properties and cytoskeleton dynamics of mDCs, which are crucial for immune function. Our finding indicated that FB1 exposure could attenuate the EPM and Young’s modulus of mDCs, indicating alterations in cell stiffness and membrane fluidity. Furthermore, FB1 could inhibit the maturation phenotypes of mDCs, including their chemotactic migration and antigen presentation ability in vitro. These functional impairments might have been caused by FB1’s interference with the change in mechanical properties and the dynamic remodeling of the cytoskeleton. Specifically, FB1 exposure significantly downregulated key cytoskeleton-binding proteins such as *CDC42*, *CAPZB* and *Cofilin*, while it upregulated *Fascin1*, suggesting a complex interplay between cytoskeletal components and FB1 toxicity. These results collectively highlight the multifaceted impact of FB1 on mDCs, linking cytoskeleton disruption to impaired immune function.

DCs are professional antigen-presenting cells derived from hematopoietic progenitor cells and present in an immature form in the skin and peripheral tissues [5]. In response to microbial products or inflammatory signals, the MHC-II, CD40, CD80, CD86 and CCR7 molecules on the surface of DCs were upregulated. CC chemokine ligand 21 (CCL21) and CCL19 are ligands for CCR7 and control the motility of DCs to the draining lymph nodes in order to induce adaptive immunity [23]. LPS, one of the most active components of the pathogen-associated molecular patterns (PAMP), can induce the maturation of BMDCs and promote the high expression of MHC-class I and II molecules, which bind a repertoire of endogenously processed peptide antigens. Then, the peptide–MHC molecule complexes are delivered to T cells, thereby initiating MHC-class I-restricted cytotoxic T lymphocyte (CTL) responses and MHC-class II-restricted Thl responses. In addition, mDCs can initiate the immune response by providing the second signals that are necessary for T cell activation through the high expression of co-stimulatory molecules, such as CD80/B7-1, CD86/B7-2 and CD40 [24]. Previous studies demonstrated that FB1 can not only inhibit the expression of co-stimulatory molecules (CD80, CD86, MHC II) and the secretions of IL-6, IL-10 and IL-12 cytokines in BMDCs [7], but also downregulate the expression of cytokines (IL-6 and IL-1β) and chemokines (CCL5 and CCL3) in human DCs [8]. In this study, after exposure to 50 μmol/L of FB1, the expression of the CD40, CD80 and CD86 co-stimulatory molecules on mDCs significant downregulated when induced with LPS. At the same time, the expression of CCR7 on mDCs was inhibited by FB1, as well as the chemotactic migration ability to CCL19. Similar to other results of the mixed lymphocyte reaction (MLR) assay [7], FB1 could reduce the ability of mDCs to trigger T cell proliferation, probably due to the reduced expression of the CD40, CD80, CD86 and CCR7 molecules that weakened the capability of migration to secondary lymphoid tissue and the activation of T lymphocytes. These results indicate that FB1 exposure might impair the immune functions of both resting imDCs and mDCs, as well as the activation of DCs.

The immune function of mDCs is closely related to their mechanical properties and cytoskeleton structure [20,25]. The present study explored the reasons why FB1 leads to the suppression of the immune function of mDCs from biomechanical and immunology perspectives, including analyses of the effects of FB1 on the mechanical properties of mDCs, such as EPM, membrane fluidity and Young’s modulus. Cell EPM is one of the biophysical parameters reflecting the charges on the cell membrane surface [26]. Cell membrane fluidity refers to the viscosity of the cell membrane or the lipid bilayer of synthetic lipid membranes, and is one of the necessary conditions for cells to perform their normal functions [27], such as the transmembrane transport of substances, intercellular and intracellular information transfer and cellular recognition. Two types of phospholipid components of the cell membrane, such as phosphatidylserine and phosphatidylinositol, are the key components of cell surface charges [28]. Some studies showed that FB1 competitively inhibits ceramide synthetase and interferes with normal sphingolipid metabolism [29], and that the rapid conversion of phosphatidylserine and phosphatidic acid can lead to changes in cell surface charges. In this study, FB1-treated mDCs showed a decrease in electrophoretic mobility (Figure 4B), supporting the hypothesis that FB1 could competitively inhibit ceramide synthase, disrupt the cell membrane structure and alter the distribution of charges on the surface of the cell membrane, which in turn correlated with the impairment of the immune function of mDCs.

In addition, the cytoskeleton, a complex and dynamic network of interlinking protein filaments extending from the nucleus to plasma membrane, is intimately involved numerous cellular physiological activities, including cell mechanics, differentiation, migration and signal transduction [12,30]. Alterations in cellular function caused by the invasion of foreign substances or the development of disease can significantly alter the mechanical properties of cells [17,31]. Consequently, the measurement of these mechanical properties can serve as a reliable indicator of cellular functions [31,32]. The present research demonstrated a substantial decrease in the cellular Young’s modulus following the depolymerization of F-actin and microtubules [33]. In this study, the effect of FB1 on Young’s modulus of mDCs was investigated and the result showed that FB1 exposure could lead to a decrease in Young’s modulus of mDCs. Consequently, the present study hypothesized that FB1 may decrease the mechanical properties and remodel the cytoskeleton of mDCs. Subsequently, the cell morphology detected by AFM scanning and immunofluorescence observation showed that FB1 could alter the cellular morphology, resulting in larger cell areas, shorter filamentous pseudopods and a decrease in F-actin content. The alteration in cytoskeletal structure may be one of the critical reasons for the impairment of the chemotactic migration and T cell activation abilities of mDCs caused by FB1. Cellular movement is powered by the assembly and disassembly of actin filaments. Among these proteins, Profilin plays a pivotal role in the dynamic reorganization of the cytoskeleton by promoting the polymerization of actin monomers (G-actin) into new actin filaments (F-actin). This process is essential for cell migration and the extension of the cell membrane. Additionally, Cofilin interacts with the Arp2/3 complex to regulate actin filament branching, while CDC42 modulates the activity of the Arp2/3 complex by activating downstream effector proteins such as N-WASP, thereby promoting actin filament branching. (CAPZB) binds to the ends of actin filaments, preventing further polymerization. The regulation of actin dynamics is orchestrated by Arp2/3 complex and the related Scar protein, capping protein, Profilin and Cofilin [34,35,36,37]. In the present study, RT-qPCR was utilized to detect the mRNA expression levels of *CDC42*, the *Arp2/3 complex*, the capping protein *CAPZB*, the actin-depolymerizing factor *Cofilin* and the monomer isolation protein *Profilin* after exposure to FB1, and the results showed that the mRNA expression levels of these cytoskeleton-binding proteins changed in different degrees. Consequently, FB1 had the potential to induce the downregulation of the cytoskeletal polymerization-associated proteins *CDC42*, *CAPZB* and *Arp2/3 complex* in mDCs. This finding suggested that the attenuated migration and immune functions of mDCs caused by FB1 may have been related to their altered mechanical properties and cytoskeletal remodeling. This may be one of the mechanisms for FB1 to exert its immunosuppressive effect.

In conclusion, the impaired migration and immune functions of mDCs induced by FB1 may be attributed to alterations in their mechanical properties and cytoskeleton remodeling. Specifically, FB1 may downregulate the expression of key cytoskeleton-associated proteins, including *CDC42*, *CAPZB*, *Arp2/3 complex* and *Cofilin*. These changes likely represent one of the mechanisms through which FB1 exerts its immunosuppressive effects.

## 4. Materials and Methods

### 4.1. Reagents and Antibodies

Recombinant murine granulocyte–macrophage colony-stimulating factor (rmGM-CSF), recombinant murine interleukin-4 (rhIL-4) and murine CC chemokine ligand 19 (CCL19) were purchased from Peprotech (Rocky Hill, NJ, USA). LPS and FITC-conjugated dextran (molecular weight: 43 kDa) were obtained from Sigma-Aldrich (St. Louis, MO, USA). APC-conjugated anti-mouse CD11c, FITC-conjugated anti-mouse CD80, CD86, CD40, PE-conjugated anti-mouse MHC-class II, (MHC-II)/CC chemokine receptor 7 (CCR7) and APC-conjugated anti-mouse CD11c antibodies were purchased from Thermo Fisher Scientific (Waltham, MA, USA). Anti-Cofilin1 antibody was from Proteintech (Chicago, IL, USA). Anti-GAPDH antibody was from HuaBio (Hangzhou, Zhejiang, China). 4′,6-Diamidino-2-phenylindole dihydrochloride (DAPI) and Cell Counting Kit 8 (CCK-8) were obtained from Solarbio (Beijing, China).

### 4.2. Animals and Ethics

The male animals (C57BL/6 mice) were obtained from the Animal Experiment Center of Guizhou Medical University and were approved by the Experimental Animal Ethics Committee of Guizhou Medical University (No. 1702104) on 6 March 2017.

### 4.3. Isolation of Bone Marrow Cells and Generation of mDCs

mDCs were generated from murine-derived bone marrow cells as described previously [38]. Briefly, bone marrow cells were flushed out from freshly dissected femurs and tibias. Following the lysis of the red blood cells in lysis buffer, the remaining cells were resuspended in RPMI 1640 (Gibco, Carlsbad, CA, USA) medium supplemented with 10% fetal bovine serum, 1% penicillin–streptomycin and differentiated into imDCs in the presence of 20 ng/mL of recombinant mouse granulocyte–macrophage colony-stimulating factor (rmGM-CSF) and 10 ng/mL of recombinant mouse interleukin 4 (rmIL-4) for 7 days. The imDCs were then matured into mDCs by adding 100 ng/mL of LPS for another 2 days. Except for the cell viability and apoptosis tests, the other analyses for mDCs were performed after treatment with 50 μmol/L of FB1 for 48 h.

### 4.4. Cell Viability and Apoptosis Assay

The cells were incubated in well plates and different concentrations (0, 10, 50 and 100 μmol/L) of FB1 were separately added. The cell viability was assessed using the CCK8 kit and apoptosis kit after being cultured at 37 °C for 48 h, which was measured using a Thermo-Scientific Microplate Reader (Bio-Rad, Hercules, CA, USA). The FACSCalibur cytometer (BD Biosciences, Franklin Lakes, NJ, USA) was used to analyze the apoptosis of FB1-treated mDCs.

### 4.5. Measurement of Cell Electrophoretic Mobility (EPM)

The 3 × 10^5^ cells were incubated in well plates with or without 50 μmol/L of FB1 for 48 h, and the cells were collected and rinsed twice with PBS. Next, a cell suspension was prepared at a concentration of 5 × 10^5^ cells/mL using a 10% (*w*/*v*) sucrose solution. The cell suspension was introduced into a cell electrophoretic migration chamber, which was mounted onto a microscope stage. Under low magnification, the static layer was identified as occupying 1/10 of the distance between the front and back wall lines of the chamber. All experimental measurements were conducted within this static layer. For each experimental group, 20 individual cells were selected, and the time required for these cells to traverse a distance of 100 μm was recorded. Measurements were performed at a temperature of 25 °C and an applied voltage of 50 V. The average time for the 20 cells was calculated, and the electrophoretic mobility of the cells was subsequently determined based on these measurements.

### 4.6. Measurement of Cell Membrane Fluidity

After treatment with 50 μmol/L of FB1 for 48 h, mDCs were rinsed twice with PBS and resuspended with RPMI-1640 (Gibco, USA) medium. Then, TMA-DPH (BestBio, Shanghai, China) was added, and the cells were incubated at 37 °C for 30 min in the dark. The cells were washed twice and resuspended with 1 mL of PBS. The absorbance was analyzed by fluorescence spectrometry. The parameters were set as follows: the excitation and emission wavelengths were 360 nm and 430 nm, the slit (EM) width was 5 nm, the excitation voltage was 400 V, and the scan time was 10 s. *p* values were calculated according to the formula of *P*= (I_VV_ − GI_VH_)/(I_VV_ + GI_VH_), G = I_HV_/I_HH_.

### 4.7. Measurement of mDCs Transmigration in Transwell

A total of 2 × 10^5^ cells in a volume of 200 μL were added to the upper chamber (5 mm pore size), and 600 μL of RPMI 1640 complete medium with 100 ng/mL of CCL19 was added to the lower chamber. The Transwell device was placed in a 37 °C incubator, and the cells were allowed to migrate for 48 h. Then, the migrated cells were counted under a light microscope.

### 4.8. Immunofluorescence

A total of 1 × 10^5^ cells were cultured on poly L-lysine-treated coverslips for 1 h and fixed in 4% paraformaldehyde for 20 min. After being permeabilized with 0.1% Triton X-100 and blocked with 1% bovine serum albumin (BSA), the cells were stained with rhodamine-phalloidin (Solarbio, Beijing, China) and DAPI in the dark for 20 min and 5 min, respectively. The cells were imaged using a laser scanning confocal microscope (Olympus or Nikon, Tokyo, Japan).

### 4.9. Real-Time Quantitative PCR (RT-qPCR) Assay

Total RNA was isolated from mDCs and then cDNA was synthesized using a reverse transcription kit (Thermo-Fisher Scientific, Waltham, MA, USA). Finally, real-time PCR analysis was carried out using specific primers (Takara, Dalian, China) to detect the expression levels of GAPDH and finally, real-time PCR analysis was performed using specific primers (Takara, Dalian, China) to detect the expression levels of GAPDH and cytoskeletal binding protein-related genes (Table 1). The formula of 2^−ΔΔCt^ was used to indicate the fold-change in the mRNA content of the target genes in the experimental group relative to the control group.

### 4.10. Mixed T Lymphocyte Reaction Experiment

T lymphocytes were isolated from mouse spleens by the nylon hair column separation method, and the cells were mixed mDCs and T cells according to the ratios (mDCs/T cells = 1:1 or 1:10), and then incubated at 37 °C in a 5% CO_2_ incubator for 48 h. Lastly, CCK8 solution was added per well, and the cells were without light for 2 h. The microplate OD_450nm_ value was recorded by Thermo Scientific Microplate Reader (Bio-Rad, Hercules, CA, USA).

### 4.11. Flow Cytometry Analysis

The cells were gathered and examined using flow cytometry under various treatment settings. Initially, the cells were treated with a 4% paraformaldehyde solution for a duration of 20 min. Subsequently, they were washed 3 times in a phosphate-buffer saline (PBS) solution for 5 min each wash. Following this, the cells were incubated with APC-conjugated anti-mouse CD11c antibodies, FITC-conjugated anti-mouse CD86/CD80/CD40 antibodies and PE-conjugated anti-mouse MHC-II antibodies and were kept on ice for incubation for 30 min. The study was conducted using flow cytometry (Beckman, Indianapolis, IN, USA) analysis after washing 3 times in PBS.

### 4.12. Atomic Force Microscopy (AFM) Analysis

A total of 1 × 10^5^ cells were cultured on poly L-lysine-treated coverslips for 1 h, and the suspension cells were washed away with PBS. Young’s modulus of the cells was measured using an atomic force microscope (AFM; JPK NanoRacer, Bruker, Billerica, MA, USA) in liquid-phase force spectroscopy mode, and the cell morphology was scanned in QI mode. For the cell morphology scanning, a gold-plated silicon nitride probe was used, and for the Young’s modulus detection, a standard silicon nitride cantilevered probe was used with a coefficient of elasticity of 0.08 N/m and a spherical needle diameter of 10 μm. The experiments were carried out under controlled conditions, in particular in a quiet and clean environment at a constant temperature of 25 °C, and Young’s modulus detection was carried out at a constant speed of 2.0 μm/s on the cells with an applied force of 1 nN. After acquiring the force and distance curves, the curves were fitted and analyzed in the range of 0–200 pN of the needle loading curve using JPK image processing software version 7.0. Subsequently, Young’s modulus of the cells was determined from the fitted curves, while the 3D morphology of the cells was obtained using JPK image processing software.

### 4.13. Statistical Analysis

Data were presented as mean ± standard deviation (SD). Each experiment was repeated at least three times, and the analysis of Student’s t test was performed to examine group differences in different variables. *p* < 0.05 was considered statistically significant.

## Figures and Tables

**Table 1:** Primers sequences of genes for RT-qPCR.

Gene Name	Primer Sequence (5’-3’)
*GAPDH*	F: ACCACAGTCCATGCCATCAC
	R: TCCACCACCCTGTTGCTGTA
*MHC-II*	F: GTGAACTGGAAGATCTTCGAGA
	R: ACTTGGTCAGTACTTTAGGTGG
*CD40*	F: GTCATAACACCGCTGCTCCAGTG
	R: TCTGTCACCTGCCGCTCCTG
*CD80*	F: CAACTGTCCAAGTCAGTGAAAG
	R: CACCACTTTGTCATGTTTTTGC
*CD86*	R: CAGCAGTCTCTGGAGTAATAGG
	R: GATTCGGCTTCTTGTGACATAC
*CCR7*	F: GATGACTACATCGGCGAGAATA
	R: ACGAAGCAGATGACAGAATACA
*CDC42*	F: CAGACTACGACCGCTAAGTTAT
	R: CAGCAGTCTCTGGAGTAATAGG
*CAPZB*	F: CTGTGTGAAGATCTCCTGTCAT
	R: GTTACTCCACGGTGACCTATAG
*Arp2/3 complex*	F: GAGACGCTCGCGCTCAAGTTC
	R: CAGCTCCATGTGCCTGAAGTTCC
*Cofilin*	F: CAGAAGAAGTGAAGAAACGCAA
	R: AGGTTGCATCATAGAGTGCATA
*Fascin1*	F: CTACTTTGACATCGAGTGGTGT
	R: CGGTTAATCAGCTTCATGAGGA
*Profilin*	F: GAAGACCTTCGTTAGCATTACG
	R: ATCCATTGTAAATTCCCCGTCT

## Data Availability

Data are contained within the article and Supplementary Materials.

## Supplementary Materials

The supporting information can be downloaded at: https://www.mdpi.com/article/10.3390/ijms26072876/s1.
