# Supplementary material for: Fumonisin B1 Exerts Immunosuppressive Effects Through Cytoskeleton Remodeling and Function Attenuation of Mature Dendritic Cells
Source: Int J Mol Sci. 2025 Mar 21;26(7):2876. doi: 10.3390/ijms26072876 (PMC11988462; doi:10.3390/ijms26072876)
Supplement: Supplementary file 1 [file ijms-26-02876-s001.zip › ijms-3498586-supplementary.pdf]

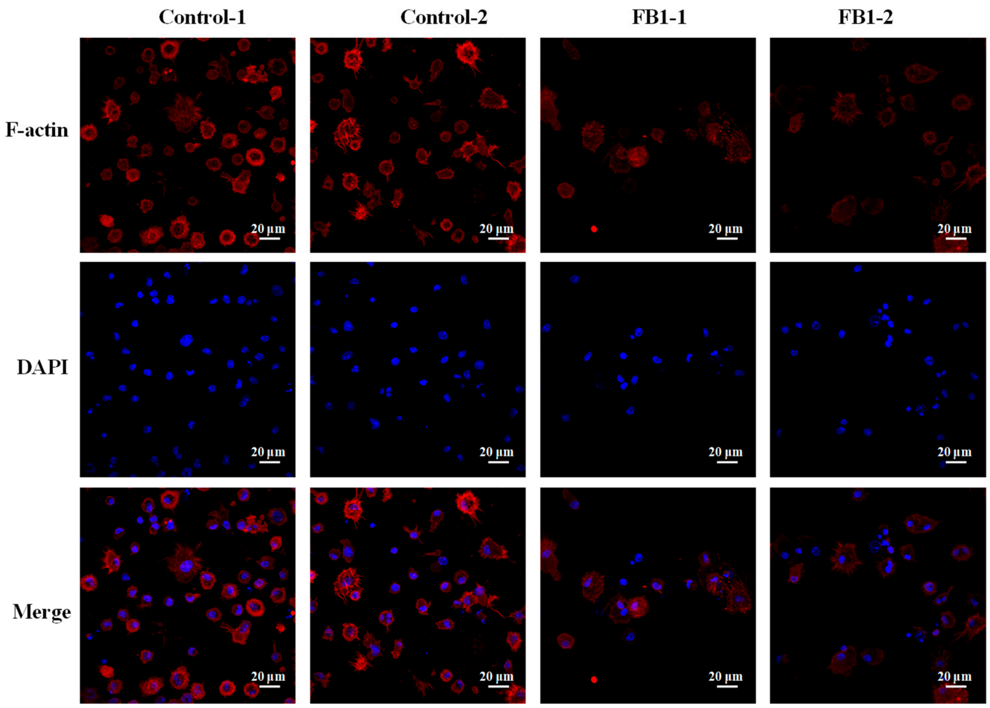

Figure S1. The cytoskeleton structure changes of mDCs induced by FB1 under Laser confocal microscopy tomography.

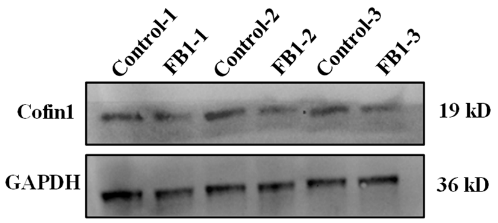

Figure S2. The protein expression levels of Cofilin1 were detected by western blot.
